# 

**Published:** 1876-02

**Authors:** 


CONTENTS:

| | PAGE |
|---|---|
| Sleeplessness | 49 |
| New York Husbands | 53 |
| Early Breakfast | 56 |
| Growing Old Happily | 57 |
| Spring Diseases | 58 |
| Failing Eyesight | 59 |
| Dangers of Oratory | 61 |
| Sources of Trouble and Joy | 62 |
| A Man | 62 |
| Successful Men | 63 |
| Sabbath Hygiene | 65 |
| Air and Exercise | 71 |
| How much to Sleep | 72 |
| Plant a Vine | 73 |
| Cancer and Constipation | 76 |
| True Hospitality | 77 |
| Teeth of Children | 78 |
| Changing Flannel | 78 |
| Water Cure | 79 |
| Over Eating | 80 |
| Averting Disease | 80 |
| Reading Aloud | 83 |
| The Best Tooth Wash | 85 |
| Corns Cured | 85 |
| Nursing | 87 |
| Liquor License | 88 |
| Gluttony | 89 |
| Bread Poison | 90 |
| Nutrition and Stimulation | 91 |
| Question of Morals | 92 |
| Bushels of Pills | 93 |
| Balance of Population | 94 |

